# Is Hydrogen Breath Test with Lactulose Feasible for Measuring Gastrocecal Transit in Critically Ill Children? Pilot Study about Modification of the Technique

**DOI:** 10.1155/2017/5878659

**Published:** 2017-01-26

**Authors:** J. López, C. Sánchez, S. N. Fernández, R. González, M. J. Solana, J. Urbano, M. Tolín, J. López-Herce

**Affiliations:** ^1^Pediatric Intensive Care Department, Gregorio Marañón General University Hospital, School of Medicine, Complutense University of Madrid, Gregorio Marañón Health Research Institute, Mother-Child Health and Development Network (Red SAMID) of Carlos III Health Institute, Spain; ^2^Gastroenterology Unit, Gregorio Marañón General University Hospital, Complutense University of Madrid, Spain

## Abstract

*Introduction*. Gastrocecal transit time (GCTT) can be measured by exhaled hydrogen after lactulose intake (lactulose-eH_2_ test). The objectives were to assess whether it is possible to carry out this test in critically ill children with and without mechanical ventilation (MV) and to analyze whether the results are consistent with clinical findings.* Methods*. Patients admitted to the Pediatric Intensive Care Unit (PICU) for more than 3 days were included. Those with gastrointestinal disease prior to admission were excluded. A modified technique to obtain eH_2_ from the ventilator tubes was performed.* Results*. Sixteen patients (37.5% boys) with a median age of 19 (5–86.5) months were included. Five patients (31.2%) were breathing spontaneously but lactulose-eH_2_ test could not be performed while it could be performed successfully in the 11 patients with MV. Seven patients (63.3%) did not show an eH_2_ peak. The other 4 showed a median time of 130 min (78.7–278.7 min) from lactulose intake to a 10 ppm eH_2_ peak. Children with an eH_2_ peak had intestinal movements earlier [6.5 (1.5–38.5) versus 44 (24–72) hours *p* = 0.545].* Conclusion*. Although the designed adaption is useful for collecting breath samples, lactulose-eH_2_ test may not be useful for measuring GCTT in critically ill children.

## 1. Introduction

Critically ill patients, both adults and children, have a high incidence of gastrointestinal motility disorders. These disorders involve an increased risk of complications: gastrointestinal (nausea, vomiting, diarrhea, or constipation), nutritional (interruptions of enteral nutrition), and nongastrointestinal (increased risk of pulmonary aspiration) [1, 2].

The pathogenesis of gastrointestinal motility disorders in critically ill patients is related to inflammatory processes, sepsis, surgery, intracranial hypertension, hyperglycemia, electrolyte disturbances, and the use of different drugs such as inotropes or opioids [1, 3–6].

Motility disturbances can affect any segment of the digestive tract: the esophagus with an increased risk of gastroesophageal reflux; the stomach causing impaired gastric emptying; the small intestine causing changes in motility patterns and poor tolerance of enteral nutrition; or the colon, with an increased prevalence of constipation [7–9].

Numerous diagnostic methods have been developed for assessing digestive tract motility in the last few decades. However, most of them cannot be used in critically ill patients due to their clinical condition, mechanical ventilation, inability to manage specific substrates with standardized preparations, or difficulties to move patients to external radiology or nuclear medicine units [10].

The measurement of exhaled hydrogen after the administration of lactulose (lactulose-eH_2 _test) has been accepted for measuring gastrocecal transit time (GCTT) in both adults and children [11–13]. Different lactulose-eH_2 _test methods in noncooperating patients have been well described and they have been used routinely by pediatric gastroenterologists [13–16].

Slow GCTT has been correlated with alterations in gut motility and gastrointestinal complications such as constipation or poor tolerance of enteral nutrition [11]. The main advantages of this test are that it is easy to perform, it is noninvasive and is of low cost, it can be performed at the patients' bedside, and it offers quick results. All these features facilitate its use in critically ill patients.

There are no validated methods for measuring GCTT in critically ill children. Nor are there any studies examining the ability of eH_2_-lactulose test to measure GCTT in critical pediatric patients.

The objectives of this pilot study were, first, to assess whether it is possible to carry out the lactulose-eH_2_ test in critically ill children with both spontaneous breathing and invasive mechanical ventilation. The second objective was to analyze whether the lactulose-eH_2_ test is able to measure GCTT in critical ill children and whether the results are consistent with clinical findings.

## 2. Methods

**Figure 1:**
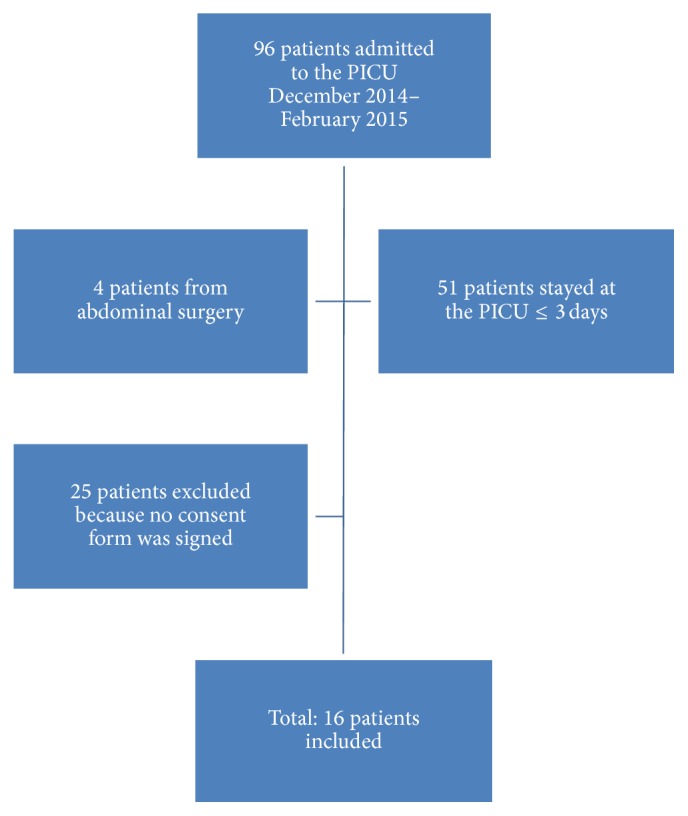
Patient recruitment flow chart.

A prospective observational study was performed in the Pediatric Intensive Care Unit (PICU) of a university children's hospital. The PICU is an 11-bed mixed medical and surgical unit with around 400 admissions per year of children between 1 month and 16 years of age. The study was approved by the Local Institutional Review Body. Patients admitted to the PICU for more than three days were offered to participate in this study. Parents or legal guardians were asked to sign the consent form. Exclusion criteria included PICU admission of less than 72 hours and known gastrointestinal disease prior to admission that could affect intestinal transit, such as abdominal surgery. This study was carried out from December 2014 to February 2015 (Figure 1).

Hydrogen monitor Gastroplus® (Isomed Pharma®, SL Madrid, Spain) was used for eH_2_ testing. This monitor measures expired hydrogen (eH_2_) in parts per million (ppm), but it does not measure methane. Before each test, monitor calibration was performed according to the instructions of the manufacturer using a standard gas sample containing 92–100 ppm eH_2_. Lactulose was used as a substrate. The oral cavity was cleaned with chlorhexidine (0.05%) before the test in every patient.

**Figure 2:**
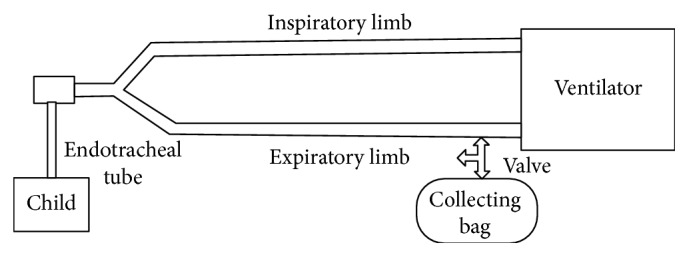
Adaptation system to allow eH_2_ measurement in patients with invasive mechanical ventilation.

An adaptation system that allowed eH_2_ measurement in patients with invasive mechanical ventilation was designed. A connection with a valve attached to a collecting bag was inserted at the end of the expiratory limb of the breathing circuit (Figure 2).

The end of the expiratory limb was the safest place for implementation because it does not alter the patient's ventilation and it does not interfere with ventilator measurements. The valve was opened at the time of measuring and several patient exhalations were collected to fill the collecting bag. Once the bag was closed, the air was removed by a 50 mL polyethylene syringe connected to a valve system to prevent losses and contamination. Then, the air was blown into the breath hydrogen monitor and eH_2_ measurement was recorded.

This adapted system was tested first in 6 healthy volunteers who were breathing spontaneously. They were asked to breathe normally, without forcing expiration, through the ventilator tubes with the inspiratory limb closed. eH_2_ measurements were obtained at baseline and 30, 60, 90, 120, 180, 240, and 300 minutes after oral lactulose intake. All of them showed normal eH_2_ measurements with peaks around 90–180 minutes after lactulose intake.

For those noncooperating children, we followed our protocol with nasobuccal masks as usual at the gastroenterology ward: our clinical practice includes a nasobuccal mask where children breathe directly connected to the hydrogen monitor. Parents press masks a bit over children faces to avoid leaks.

Lactulose-eH_2 _tests were made a few days after PICU admission and in children under no antibiotic treatment. Postsurgical children had only one cefazolin dose just before surgery starting so effect over the test was considered almost zero. We obtained baseline eH_2_ measurements from those patients included in the study before we administered 0.5 g/kg of oral lactulose (maximum dose 10 g) diluted to 10% water by the nasogastric tube. After that, eH_2_ measurements were performed every 30 minutes for the first 2 hours and every 15 minutes for the following 4 hours or until a peak rise in breath hydrogen by 10 ppm above baseline was reached. Patients without an eH_2_ peak after 6 hours were considered non-hydrogen producers.

Collected data included age, sex, weight, history of constipation (defined as hard stools and/or a frequency of less than 1 bowel movement every 48–72 hours), diagnosis, reason for admission, illness severity scores at admission (Pediatric Risk of Mortality III [PRISM III], Pediatric Index of Mortality 2 [PIM2], and Pediatric Logistic Organ Dysfunction [PELOD]) [17–19], and length of PICU stay. Constipation was defined as an absence of bowel movements for more than 3 days based on criteria used in critically ill adult and pediatric patients [3, 4, 6, 20, 21]. A daily record of bowel movements, doses of intravenous sedative, analgesic, muscle relaxant, and inotropic drugs, and the need for mechanical ventilation was kept throughout the patient's PICU admission (up to 7 days). The starting day of enteral nutrition as well as the number of episodes of vomiting and the presence of abdominal distension were also recorded.

SPSS 21.0 software package (IBM SPSS Statistics, Chicago) was used for data analysis. Absence of normality was assured with the Kolmogorov-Smirnov test. Continuous variables are expressed as medians (interquartile range, IQR) and categorical variables as percentages. Mann–Whitney *U* test and Fisher exact test were used for comparisons between continuous and categorical variables, respectively. Statistical significance was taken as a *p* value of less than 0.05.

## 3. Results

Sixteen patients (37.5% boys) were included in this study. The median age was 19 (5–86.5) months and median weight was 7.2 (5.2–18.7) kg with a median length of stay of 15 (3.7–25.7) days. Constipation prior to admission was reported in 37.5% of patients. The most frequent cause of admission was postoperative cardiac surgery (13 patients: 81.3%). Other causes were respiratory failure, neurologic disease, and neurosurgery (1 patient each: 6.3%). The median scores on clinical severity scales were PRISM III 10.0 (1.5–30.8), PIM2 2 (0.3–16), and PELOD 9.2 (0.1–19.6).

Five patients (31.2%) were breathing spontaneously. Two of them were excluded from the study: one vomited after lactulose intake and the other one had breakfast 90 minutes after lactulose administration. In the other three patients, the test had to be interrupted before any results were obtained due to the anxiety that the expiration through the mask for eH_2_ measurement was causing.

Eleven patients with invasive mechanical ventilation were studied and eH_2_ test was performed successfully in all of them. The median age was 16 months (4–90 months) and median weight was 7.1 kg (5.1–19 kg). The most frequent cause of admission was postoperative cardiac surgery (8 patients: 72.3%). Other causes were respiratory failure, neurologic disease, and neurosurgery (1 patient each: 9%). The median scores of clinical severity scales were PRISM III 1.10 (1.5–33), PIM2 7.6 (0.8–17.8), and PELOD 16.2 (0.1–20.8). Constipation prior to admission was reported by parents in three patients (27.3%).

The lactulose-eH_2_ test was performed after a median of 5 days (2–9 days) of PICU admission. Three patients were fasted at the time of the test, while the rest of them were on continuous enteral nutrition through a nasogastric or transpyloric tube. The median time to start intestinal movements was 4 days (2–5 days). Six patients (54.5%) presented constipation during PICU stay.

Seven patients (63.3%) did not show an eH_2_ peak during the 6-hour study after lactulose intake. In the other four patients, median time from lactulose intake to a 10 ppm eH_2 _peak was 130 min (78.7–278.7 min) (normal time in healthy children is 65 ± 15.3 minutes) [12, 22].

There were no differences in any of the variables between children with and without an eH_2_ peak (Table 1).

Children with an eH_2 _peak had intestinal movements earlier than those without one [6.5 (1.5–38.5) versus 44 (24–72) hours], although no significant differences were found (*p* = 0.545).

There were no side effects from lactulose intake or from the modified technique to obtain eH_2_ from the ventilator tubes.

## 4. Discussion

Our study is the first to analyze the possibility of using eH_2_ breath test to measure GCTT in critically ill children. Lactulose-eH_2 _test is based on the fact that this substrate is a nonabsorbable disaccharide that is fermented by colonic bacteria. The hydrogen produced by the bacteria is absorbed through the wall of the small or large intestine, or both. The hydrogen-containing blood travels to the lungs where the hydrogen is released and exhaled in the breath where it can be measured [13]. eH_2_ measurements are made every 10–15 minutes for a period between 180 and 240 minutes. GCTT is the time between the administration of lactulose and the rise of at least 10 ppm of eH_2_ from baseline in two followed determinations. The test has a better reproducibility for solid lactulose, but administration is more complicated in critically ill patients, so a liquid solution was used in our study.

Several studies found a good correlation between prolonged GCTT measured with lactulose-eH_2 _test in constipated children [13, 22] while other studies did not [23]. The main limitation of this test is the variability of results in healthy volunteers and the lower reproducibility of the test with the liquid substrate [10, 13]. Other researchers have also faced difficulties in interpreting eH_2_ breath test [24].

Our study shows that lactulose-eH_2 _test is not feasible in critically ill children with spontaneous breathing. This test requires cooperation that is almost impossible to obtain due to their clinical situation: either they are unable to perform forced active expiration or this generates anxiety. Stress and hyperventilation in 3 out of 5 patients made it impossible to perform the test correctly.

In mechanically ventilated adults, eH_2_ measurement is connected directly to the endotracheal tube [25]. However, this can greatly increase respiratory dead space and produce significant hypoventilation in children. Therefore, it is necessary to collect the air in a special collecting bag, so a T-tube with a control valve was inserted in the expiratory limb of the breathing circuit (Figure 2) to reduce handling of the endotracheal tube. This allowed an adequate minute volume with no impact on ventilation parameters. The test in healthy volunteers showed that this method is suitable for collecting samples for testing.

A 10 ppm eH_2_ increase was absent in most of our patients and, in patients in whom it was present, no correlation was found between GCTT and patient's defecation rhythm or any other clinical or treatment variables.

GCTT could only be measured in four patients (36.4%), and although it was longer for constipated children [22], no correlation was found between the GCTT and the time to stooling or any other nutritional complication, suggesting that the test has no clinical usefulness in the critically ill child.

The failure to detect eH_2_ levels in a high percentage of patients could be due to several factors [24]. First, these children could be methane producers instead of hydrogen producers. Methane was not measured in our study. The percentage of methane production in the general population varies between 5 and 34% [10, 24]. However, this percentage is much lower than the failure to detect eH_2_ observed in our study (64%). Another possible factor might be a very slow GCTT (of more than 6 hours). Some drugs such as inotropes or opioids [26] slow GCTT, but no significant differences in doses of inotropes or opioids were found between children with positive and negative eH_2 _test in our study. There was no correlation with a delay in intestinal movements either. Moreover, lactulose administration directly into the stomach through a nasogastric tube could justify slight acceleration but not a delay in GCTT.

Therefore, according to our results, lactulose-eH_2_ test does not seem to be useful to measure GCTT in children with mechanical ventilation, although the designed amendment is useful for collecting breath samples. Moreover, given the high number of measurements that must be obtained to determine GCTT over 6 or more hours, this test would determine a significant burden of work for the nursing staff.

There are other devices for measuring GCTT in constipated children as ^13^C-lactose, colonic radioscintigraphy, colonic manometry capsule motility, or radiopaque markers. However, most of these techniques cannot be performed in critically ill patients due to invasiveness, the need for patient mobilization, the lack of reference values, the need for endoscopy, or the difficulty of managing radiotracers [10, 27–30].

Our study has certain limitations due to the low number of patients, as the study was terminated prematurely due to the absence of positive results. Nevertheless, the number of subjects was sufficient to meet the first objective of the study. More studies about this should be done to clarify this item.

## 5. Conclusions

Although the designed adaption is useful for collecting breath samples, lactulose-eH_2_ test seems to be of little utility for measuring GCTT in critically ill children because of the high amount of work and difficulties associated with it.

## Figures and Tables

**Table 1:** Comparison between patients with positive and negative expired H_2_ peak.

Variable	No eH_2_ peak, *n* = 7 (63.6%)	eH_2_ peak, *n* = 4 (36.4%)	*p* value
Demographic data at admission			
Age (months)	22 (4–102)	13.5 (5–61)	0.545
Weight (kg)	6.2 (5.1–26)	7,2 (4.5–7.8)	1
Males	2 (28.5%)	1 (25%)	0.721
Previously constipated	2 (28.5%)	1 (25%)	0.721
Cardiac surgery	6 (85.7%)	2 (50%)	0.279
Clinical severity scores			
PRISM III (%)	24.5 (1.5–53.4)	5.4 (1.2–10.5)	0.061
PIM2 (%)	4.9 (0.8–64.4)	11.2 (2–16)	1
PELOD (%)	16.2 (0.1–20.8)	0.7 (0.1–12.5)	0.236
Vasoconstrictors (epinephrine/norepinephrine)	1 (14.2%)	1 (25%)	0.618
Sedation/analgesia			
Midazolam (mcg·kg^−1^·min^−1^)	2 (0–3)	0.35 (0–0.9)	0.194
Fentanyl (mcg·kg^−1^·h^−1^)	2 (1–3)	0.6 (0-1)	0.194
Muscle relaxants (vecuronium)	2 (28.5%)	0 (0%)	0.382
Gastrointestinal			
Fasting	2 (28.5%)	1 (25%)	0.721
Vomiting	1 (14.2%)	0 (0%)	0.636
Constipated patients	3 (42.8%)	3 (75%)	0.348
Length of PICU stay (days)	15 (11–36)	22 (6.2–44.5)	1
Time from admission to lactulose-eH_2_ test (days)	4 (1–9)	7.5 (2.25–16.5)	0.242

H_2_: hydrogen; PRISM III: Pediatric Risk of Mortality III; PIM2: Pediatric Index of Mortality 2; PELOD: Pediatric Logistic Organ Dysfunction; PICU: Pediatric Intensive Care Unit.

Continuous variables are expressed as medians (interquartile range) and categorical variables as absolute number (percentage).
